# A New Treatment Strategy for Lung Cancer With HDAC and Wnt/ *β* ‐Catenin Pathway Inhibitors

**DOI:** 10.1002/iub.70037

**Published:** 2025-07-12

**Authors:** Elif Erturk, Oguzhan Akgun, Yaren Yildiz, Gonca Tuna, Ferda Ari

**Affiliations:** ^1^ Vocational School of Health Services Bursa Uludag University, Bursa Turkiye; ^2^ Department of Biology, Science and Art Faculty Bursa Uludag University, Bursa Turkiye

**Keywords:** apoptosis, cell cycle, cell migration, cytotoxicity, EMT, lung cancer, transition

## Abstract

Lung cancer is a type of cancer with high morbidity and mortality rates worldwide. The overall survival rate of lung cancer patients is low due to a lack of therapeutic options. Recently, the combination of histone deacetylase (HDAC) inhibitors with anti‐cancer agents offers a promising therapeutic strategy for cancer treatment. Repurposing these drug combinations is important to evaluate their preventive effect on the epithelial mesenchymal transition (EMT) phenotype, which plays a critical role in tumor progression and metastasis. In this study, the changes that the combination of the HDAC inhibitor Valproic acid (VPA) and Wnt/β‐Catenin pathway inhibitor Niclosamide (Niclo) may cause in cytotoxicity, apoptosis, cell cycle, and EMT mechanisms in lung cancer cell lines (A549 and H1299) were examined. According to the results, the combination of VPA + Niclo significantly reduced cell viability in lung cancer cells compared to the use of Niclo alone. ELISA and Western blot analyses revealed that the combination of VPA + Niclo significantly enhanced the total acetylation of Histone H3 compared to the use of VPA alone. It was also found that the combination treatment induced apoptosis by increasing the activity of Caspase 3/7 and Annexin‐V and significantly increased the percentage of apoptotic cells by causing depolarization of mitochondria. After cell cycle analysis, the combination treatment increased G1 phase retention in A549 cells, while G1‐G2/M phase retention increased in H1299 cells. Wound healing and transwell migration assay results showed that the VPA + Niclo combination treatment inhibited cell migration in lung cancer cells. According to Western blot and PCR results, after VPA + Niclo treatment, the increase in E‐Cadherin levels and the decrease in *β*‐Catenin, Fibronectin, Vimentin, and N‐Cadherin levels at both protein and gene levels indicated that combination therapy may be useful in preventing the EMT process in lung cancer cells. As a result of the analyses, it was seen that VPA + Niclo combination therapy could play a critical role in preventing the acquisition of the mesenchymal phenotype, reducing cell migration and invasion ability, and preventing tumor cell survival and resistance to apoptosis. In conclusion, it was determined that VPA + Niclo combination treatment shows anticancer activity in lung cancer cells and is a promising approach that may have a synergistic effect in inhibiting EMT.

## 1 Introduction

Lung cancer is the second most common type of cancer worldwide after breast cancer, and it ranks as the first leading cause of cancer‐related deaths. According to the latest data, it is estimated that there were about 2.21 million new cases of lung cancer in 2020 [1, 2]. Five‐year survival rates are 59% for stage 1/2 lung cancer, 31.7% for stage 3, and this rate unfortunately decreases to 5.8% in the metastatic patients [3]. These data show that metastasis in lung cancer is the biggest obstacle to treatment and patient survival.

One of the main mechanisms of metastasis is Epithelial Mesenchymal Transition (EMT), which triggers the migration capability of cancer cells. Not only in metastasis, EMT contributes to cancer progression by playing a role in drug resistance in most cancer types, and, moreover, EMT‐derived cells may have similar properties to cancer stem cells [4, 5]. Changes in epithelial and mesenchymal cell markers, transcription factors such as Snail, Twist, and ZEB, matrix metalloproteinases, various signaling pathways (including TGF‐*β*, Wnt, Notch and Hedgehog), and epigenetic factors are involved in the induction of EMT [5, 6].

Epigenetics are changes in gene expression that do not result from altering the DNA sequence, but occur through various mechanisms at the DNA and chromatin level [7]. DNA methylation, histone acetylation‐deacetylation/methylation/phosphorylation/ubiquitination are the major epigenetic modifications in the regulation of EMT in lung cancer [8]. Many studies to date have shown that new treatment approaches targeting these mechanisms with epigenetic agents such as histone deacetylase (HDAC) inhibitors and DNA methylation inhibitors are of great importance in order to prevent lung metastasis [8, 9, 10, 11, 12]. In addition to EMT inhibition, HDAC inhibitors may exert pleiotropic effects on cancer cell death, cell differentiation, angiogenesis, cell cycle, and immune modulation in lung cancer [13, 14].

So far in preclinical studies, many compounds as HDAC inhibitors have been developed, such as VPA (Valproic acid), MS‐275 (entinostat), FK228 (romidepsin), SAHA (suberoylanilide hydroxamic acid), Trichostatin A, LBH589 (panobinostat), and PXD101 (belinostat). Among these, there are HDAC inhibitors that have achieved successful clinical results in different cancer types such as myeloma and T‐cell lymphoma, alone or in combination treatment [15, 16, 17]. VPA (Class‐1 HDAC inhibitor), one of the most common HDAC inhibitors, is a Food and Drug Administration (FDA) approved drug primarily used for the treatment of epileptic seizures [18]. Evidence collected so far proves that VPA has anti‐tumor activity in many types of cancer by suppressing cell proliferation, affecting cell cycle and cell death, and inhibiting cell migration and invasion [19, 20, 21, 22, 23]. In addition, VPA has been shown to inhibit EMT in renal cell carcinoma, breast cancer, and prostate cancer [24, 25]. Although VPA is considered a promising epigenetic agent, its combination with different drugs seems more effective; therefore, combination studies have been strongly emphasized in recent years [23, 26, 27].

Niclosamide (Niclo) is an FDA‐approved drug that has been used as an anthelmintic in humans for over 50 years; it has also been shown to have anti‐tumor potential in different types of cancer [28, 29]. It targets Wnt/*β*‐catenin, STAT3, NF‐κB, mTORC1, Notch pathways, and mitochondria to induce cell death; thus, it has become a remarkable agent in cancer research with the property of multiple pathways inhibitor [29, 30]. For this reason, combination studies of Niclo indicate that its antitumor effect can be enhanced [29, 31].

Alternative drug development studies, which are common for multiple pathways of cancer, are one of the key points of treatment research today. The cost of existing treatments, their various side effects, and the development of resistance of cancer cells to chemotherapy cause a demand for new drugs. Drug repurposing is a new approach that focuses on finding new applications for old clinically approved drugs. With this approach, the original indication and mechanism of action of each repurposed drug in cancer are identified. Clinical trials are vital to determining drug safety and effectiveness, resulting in data demonstrating the antitumor effectiveness of anti‐cancer drugs and compounds in drug classes. While some induce cancer cell death or suppress aspects of cancer cell behavior in established tumors, others may prevent cancer development. On the other hand, improving the effect of current drugs or revealing new mechanisms of action can provide advantages in many aspects, such as therapeutic response and cytotoxicity concerns, determination of pharmacokinetic profiles, time and cost, compared to new drug development studies [32]. In this context, in our previous study, we showed that the combination of VPA and Niclo significantly reduced cell viability in the A549 lung cancer cell line and induced cell death via the extrinsic apoptotic pathway [33]. Based on the vital role of EMT in treatment and patient survival, the aim of our current study was to evaluate the effect of VPA + Niclo combination on EMT in A549 and H1299 cells.

## 2 Materials and Methods

### 2.1 Cell Culture

The H1299 and A549 human lung cancer cell lines were obtained from ATCC (American Type Culture Collection) and was cultured in RPMI 1640 (Gibco, Grand Island, NY, USA) supplemented with 100 U/mL penicillin + 100 μg/mL streptomycin (Gibco, Grand Island, NY, USA), 2 mM L‐glutamine (Gibco, Grand Island, NY, USA), and 10% fetal bovine serum (Lonza, Verviers, Belgium), at 37°C under 5% CO_2_. Niclo and VPA were purchased from Sigma‐Aldrich (Catalog nos: N3510, P4543; Sigma‐Aldrich, MO). Stock solutions of VPA and Niclo were prepared as described before [21].

### 2.2 Determination of Cell Viability by Adenosine 5′‐Triphosphate Assay

The adenosine 5′‐triphosphate (ATP) viability assay, which is a highly sensitive method, is based on the principle of determining viability by measuring the metabolic function of cells (detection of the ATP produced by living cells). For the ATP assay, H1299 cells were seeded at 5 × 10^3^ cells/well in a 96‐well plate. VPA (0.07‐5 mM) and Niclo (0.07‐5 μM) were applied at different concentrations and incubated for 48 h at 37°C under 5% CO_2_. For combination therapy, 500 μM VPA was pre‐treatment for 48 h followed by 24 h of Niclo (0.07‐5 μM) treatment. At the end of the incubation period, the ATP assay was performed as described in the previous study [34]. Cytotoxicity analyses were performed and evaluated at different time periods. No statistically significant difference was detected between 48 h of VPA treatment followed by 48 h of Niclo treatment and Niclo alone (Figure S1). The 48 h treatment duration was selected based on preliminary time‐course optimization studies and supported by previous literature. Our preliminary studies showed that the most pronounced effects were observed at the 48 h time point, supporting its use as the optimal treatment duration [33].

### 2.3 Acetylated Histone H3 Level

Determination of total histone H3 acetylation was performed using an ELISA kit (PathScan Acetylated Histone H3 Sandwich ELISA kit, #7232; Cell Signaling Technology, Danvers) based on the principle of measuring acetylated lysine levels. H1299 cells were treated with VPA and Niclo alone and in combination. The analysis was performed following the steps in the kit protocol.

### 2.4 Apoptosis Analysis by Flow Cytometry

To investigate the apoptotic pathway, caspase 3/7 activity, Annexin‐V staining, and mitochondrial membrane polarization measurement were performed by flow cytometry using the relevant kits (Muse caspase 3/7 kit, #MCH100108, Millipore; MuseMito Potential Assay Kit, #MCH100110, Millipore). H1299 cells were seeded in six‐well plates (3 × 10^5^ cells per well) and treated with VPA and Niclo alone and in combination as described above. At the end of the incubation period, analyses were performed according to the kit protocol.

### 2.5 Cell Cycle Analysis

Cell cycle analyzes were performed by flow cytometry. A549 and H1299 cells treated with VPA and Niclo alone or in combination, and untreated control cells were washed with PBS and fixed with 70% ethanol. After the fixation, the cells were washed with PBS again, and then the Cell Cycle Reagent was pipetted and incubated for 30 min in the dark. At the end of the incubation period, cell cycle distribution analysis was performed with the Muse Cell Analyzer.

### 2.6 Colony Formation Assay

A549 and H1299 cells were seeded in 6‐well plates at 500 grains per well. Cells were treated with VPA (500 μM) and Niclo (5 μM), alone and in combination. At the end of the 48 h treatment period, the wells were replaced with drug‐free/fresh medium every 3 days, and the growth of the cells was examined under an inverted microscope day by day. When 50 cells per colony were formed in the cells in the control group, the medium in the wells was removed and fixed by adding 1 mL of cold methanol to each well. In order to remove the methanol, they were washed once with 1X PBS and then incubated with 0.05% crystal violet for 20 min in the dark to allow staining. At the end of the period, 1X PBS was washed once again, and then images of the cells were taken under the microscope with 4× and 10× objectives. The change in the colony density depending on the dose was analyzed with the Image J computer program in the images [35, 36].

### 2.7 Wound Healing Assay

A549 and H1299 cells were seeded in 6‐well plates at 15 × 10^4^ cells per well. When 90% filling was reached, vertical lines were created in the wells with a 1000 μL pipette tip. Then, VPA 500 μM, Niclo IC_70_ dose (0.3 μM dose) and VPA + Niclo combination treatment were applied, while their own medium was added to the cells in the control group. Niclo was used at 0.3 μM in migration assays to avoid cytotoxic effects observed at 5 μM, which could interfere with cell motility analysis. This lower, sub‐cytotoxic dose allowed accurate evaluation of migration without compromising cell viability. Images of wound‐forming cells were obtained with an inverted microscope at 12 h intervals until the gap between the cells in the control group was completely closed. To quantitatively measure wound healing, the distance between the cells in the control group was taken as a reference, and the closure rate in the treated wells was calculated with the Image J computer program [37].

### 2.8 Transwell Migration Assay

Transwell membranes were placed in 24‐well cell culture dishes, and 1 × 10^5^ A549 and H1299 cells were added in 100 μL volumes. VPA 500 μM, Niclo IC_70_ dose (0.3 μM dose), and VPA + Niclo combination treatment were applied to the cells. Finally, 20% FBS‐containing medium (chemoattractant) was added to the outside of the transwell and incubated in a 37°C incubator. After 48 h of treatment, cells were fixed using 70% ethanol, and the cells that migrated to the bottom of the transwell were stained with 0.2% crystal violet dye. Photographs of the stained cells were taken using a phase contrast microscope, and cell migration was calculated using Image‐J software.

### 2.9 Protein Analysis by Western‐Blotting

For Western blot analysis, A549 and H1299 cells were seeded into 6‐well plates; VPA (500 μM) and Niclo (5 μM) treatment alone and in combination were applied. Cells were lysed using RIPA Lysis Buffer System (Santa Cruz Biotechnology, Dallas) and protein content was obtained. Protein amounts of the samples were determined by bicinchronic acid assay (BCA, Thermo Fisher Scientific). Equal amounts of protein for each sample were loaded onto 10% SDS‐polyacrylamide gels and subjected to electrophoresis. Western blot steps were performed as described previously [33]. Fibronectin (#26836), Vimentin (#5741), E‐Cadherin (#3195), N‐Cadherin (#13116), Acetyl‐Histone H3 Lys9 (Ac‐H3k9, #9649), Glyceraldehyde 3‐Phosphate Dehydrogenase (GAPDH, #5174) primary antibodies obtained from Cell Signaling Technology were used. Visualization was performed using Fusion FX‐7 imaging device system.

### 2.10 Real‐Time Quantitative Polymerase Chain Reaction (RT‐qPCR) Analysis

To obtain RNA samples from A549 and H1299 cells, the PureLink RNA Mini Kit (Thermo Fisher Scientific, CA) was used in accordance with the manufacturer's instructions. RNA quality was assessed using the NanoDrop 2000 (Thermo Fisher Scientific), and cDNA synthesis was carried out with the High‐Capacity cDNA Reverse Transcription Kit (Thermo Fisher Scientific). RT‐qPCR analysis was then performed using the primers listed in Table 1 on the Applied Biosystems Step One Plus Real‐Time PCR (Thermo Fisher Scientific). Each experiment was conducted in triplicate and repeated twice. The gene expression data was compared to the negative control (untreated group) and statistical significance was set at a minimum of two‐fold increase. The resulting graph depicts the comparison of the obtained data to the negative control group. The SYBR Green method was used for Ct detection.

**TABLE 1:** The primer sets for real‐time polymerase chain reaction.

Genes	Forward	Reverse
ABCC1	5‐GACCGAGGCTACATTCAGATG‐3	5‐TCCCAGAAAGAGTAGAAGAGGT‐3
ABCB1	5‐CCCTTGTTAGACAGCCTCATATT‐3	5‐GCTTTGTCCAGGGCTTCTT‐3
FN1	5‐CATCGAGCGGATCTGGCCCC‐3	5‐GCAGCTGACTCCGTTGCCCA‐3
VIM	5‐TGCAGGAGGCAGAAGAATG‐3	5‐TCCGGTACTCAGTGGACTC‐3
CDH1	5‐CCTGCCAATCCCGATGAAA‐3	5‐CATAGTCAAACACGAGCAGAGA‐3
CDH2	5‐CCATCAAGCCTGTGGGAATC‐3	5‐GCAGATCGGACCGGATACTG‐3
SMAD2	5‐ATGTCGTCCATCTTGCCA‐3	5‐AACCGTCCTGTTTTCTTTAG‐3
SNAII	5‐CTCTTTCCTCGTCAGGAAGC‐3	5‐GCTGGAAGGTAAACTCTGGATTA‐3
CTNNB1	5‐GTATGAGTGGGAACAGGGATTT‐3	5‐TGAGCTCGAGTCATTGCATAC‐3
GAPDH	5‐GCTCTCTGCTCCTCCTGTTC‐3	5‐ACGACCAAATCCGTTGACTC‐3

### 2.11 Statistical Analyses

The results of the experiment were reported as mean ± standard deviation (SD), which was calculated by averaging data from at least three independent studies. Statistical analyses were done using GraphPad Prism 8.0 (Demo Version; GraphPad, San Diego, CA) and one‐way analysis of variance was used to determine significance. Statistical significance was set at *p* < 0.05, *p* < 0.01, and *p* < 0.001 for the analyses.

## 3 Results

### 3.1 Antigrowth Effect Combination Therapy of VPA + Niclosamide

**FIGURE 1:**
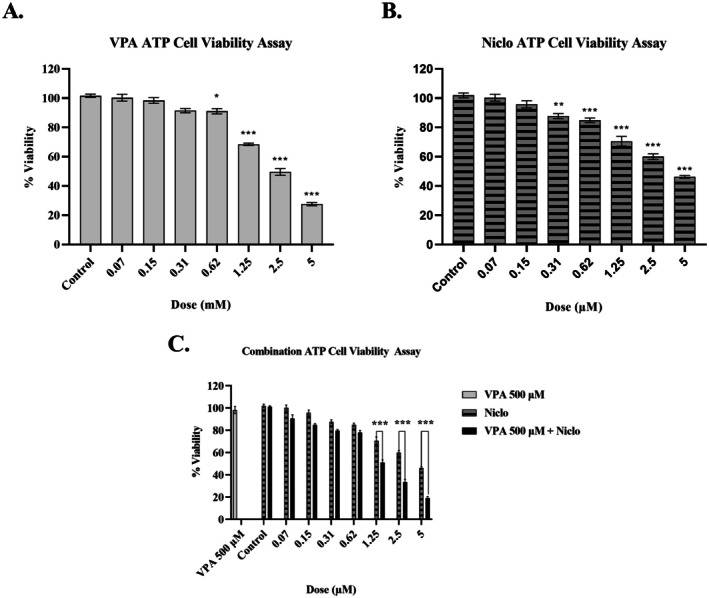
Percent viability graphs of H1299 cell treated with (A) Valproic acid (VPA) (0.07–5 mM), (B) Niclosamide (Niclo) (0.07–5 μM), and (C) VPA (500 μM) + Niclo (0.07–5 μM) for 48 h. ATP assay was performed as cell viability assays. *Denotes statistically significant differences compared to untreated control: *p < 0.05, **p < 0.01, ***p < 0.001. Data are presented as mean ± SD (*n* = 3).

The growth inhibitory effects of VPA (0.07–5 mM) and Niclo (0.07–5 μM) on cells were evaluated after 48 h. In the results of the ATP assay, it was observed that the treatment of VPA and Niclo for 48 h in H1299 cells decreased cell viability in a dose‐dependent manner (Figure 1A,B). Accordingly, combination therapy was performed using different concentrations of Niclo (0.07–5 μM) in combination with a non‐toxic dose of VPA (500 μM). As a result, the VPA + Niclo combination significantly reduced cell viability in H1299 cells compared to Niclo alone at doses of 1.25, 2.5, and 5 μM (Figure 1C) (*p < 0.05, **p < 0.01, ***p < 0.001). In our previous study, it was determined that the combination therapy did not have a statistically significant effect on BEAS‐2B cells, which are healthy lung cells [33].

The specific dose selection to be used in further analyses for the A549 cell line was determined through a study involving the same experimental set that our team had previously performed [33]. According to the ATP cell viability method, the calculated IC_50_ value for 48 h VPA treatment is 2.53 mM, for Niclo treatment is 4.32 μM and for VPA + Niclo combination treatment is 1.41 μM concentration. IC_50_ values (the concentration of the compounds that inhibit the response by 50% compared to control cells) are given in Table 2.

**TABLE 2:** IC_50_ values of H1299 cells treated with VPA, Niclo, and VPA + Niclo according to ATP assay results.

Compound	H1299 IC_50_ (μM)	A549 IC_50_ (μM) [33]
VPA	2.53 mM	> 5 mM
Niclo	4.32 μM	1.74 μM
VPA + Niclo	1.41 μM	2.34 μM

*Note:* IC_50_ is defined as the dose inhibiting 50% of viability.

**FIGURE 2:**
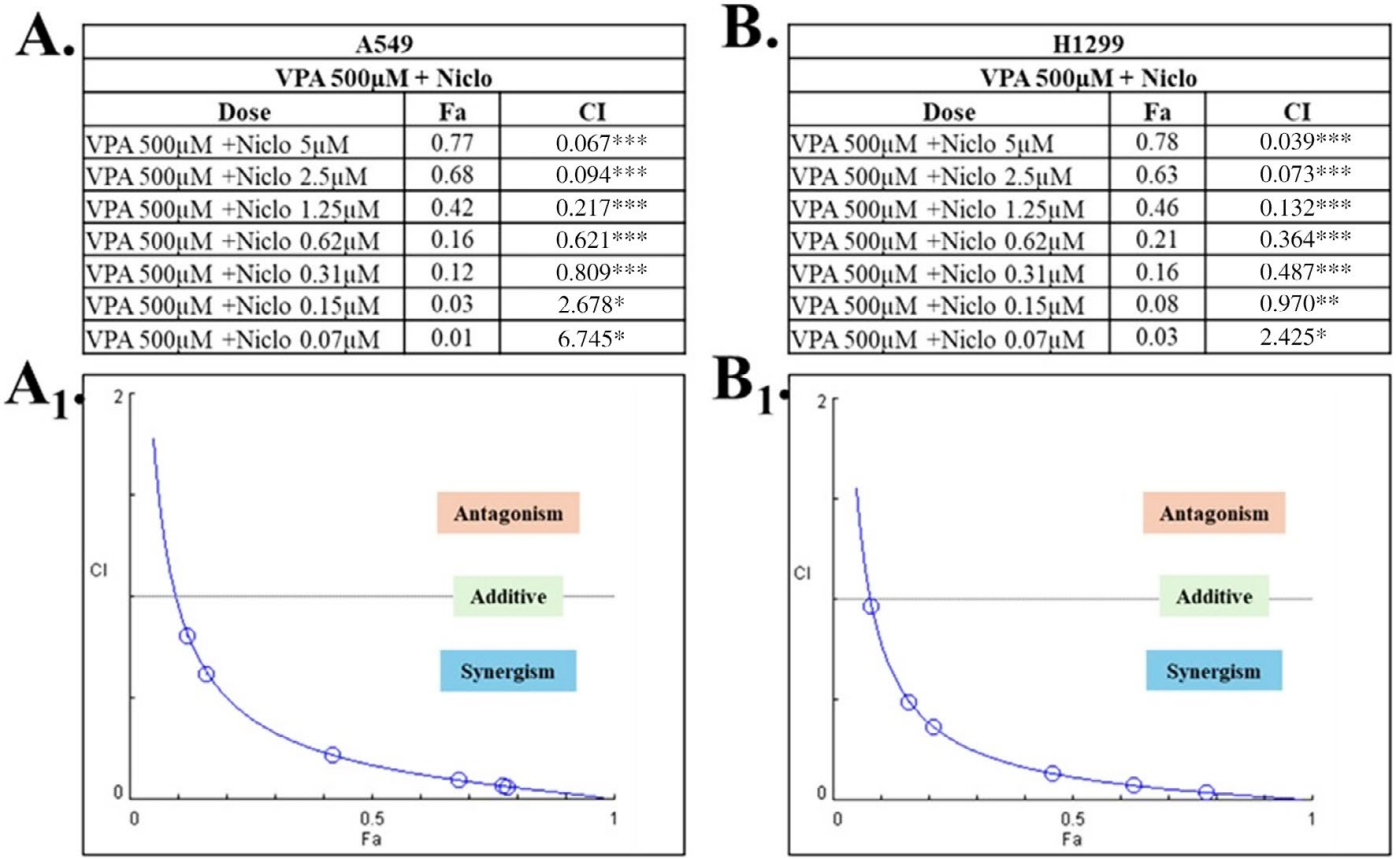
CompuSyn CI index results of VPA (500 μΜ) and Niclo (5 μΜ) combinations in A549 and H1299 cell lines. VPA and Niclo combination shown in (A) A549, (B) H1299. CompuSyn CI index results for VPA and Niclo combination; (A1) A549, (B1) H1299. *According to the CI value in combination; ***CI < 0.9 Synergism, **0.91.1 Antagonism.

Furthermore, according to the CompuSyn analysis, however, the majority of the combination doses used showed synergistic effects (CI < 0.9). The highest synergistic effect was observed in the combination of VPA (500 μM, 48 h) and Niclo (5 μM, 24 h) (Figure 2). According to our cytotoxicity and CombuSyn results, VPA 500 µM and Niclo 5 µM doses, which were both synergistic and cytotoxic, were used as combination doses in further analyses.

### 3.2 Combination of VPA + Niclosamide Induces Histone H3 Acetylation

**FIGURE 3:**
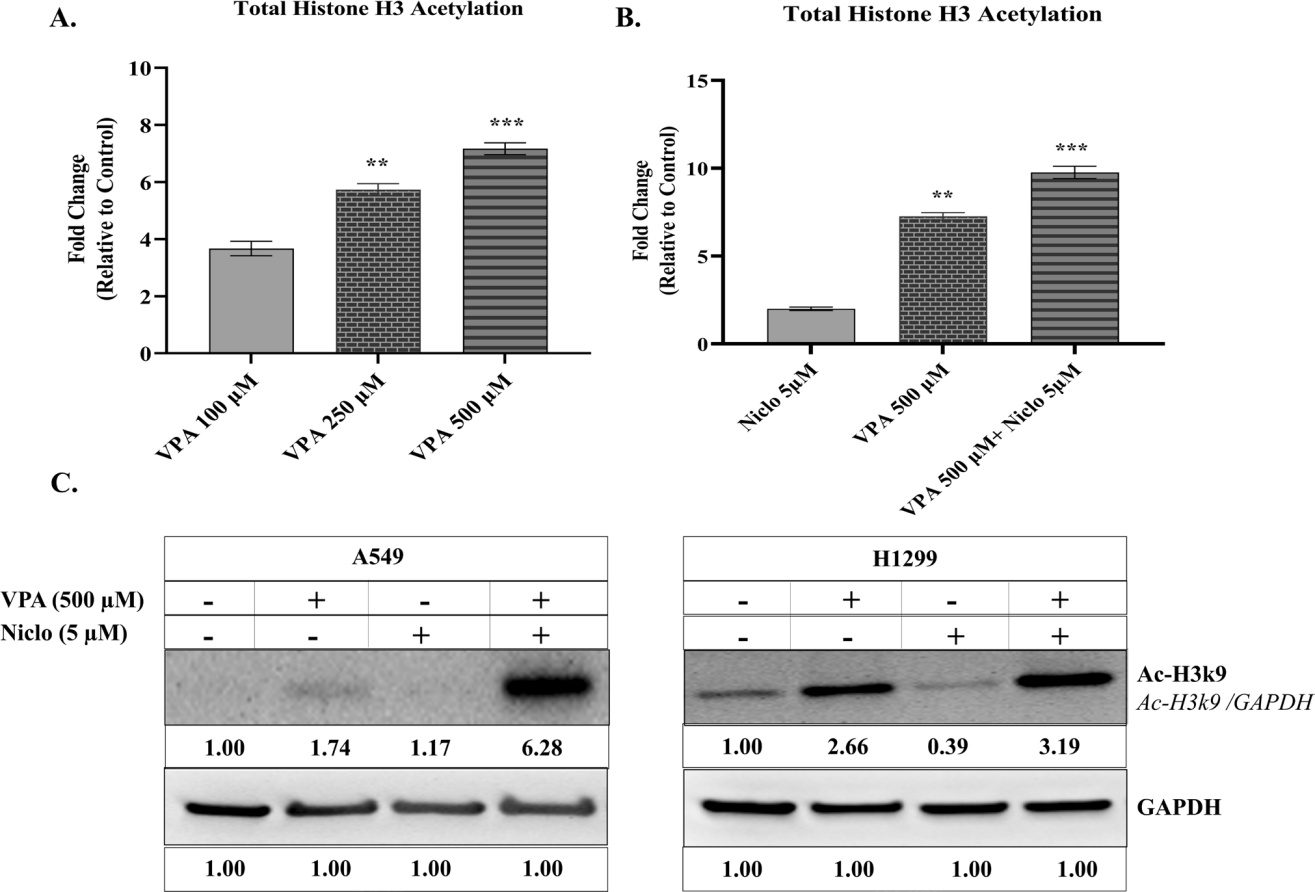
(A) Levels of H3 acetylation occurring in H1299 cell after 48 h of Valproic acid (VPA) (100–500 μM) treatment. (B) Levels of H3 acetylation occurring in H1299 cell after 48 h of VPA (500 μM), Niclosamide (Niclo) (2.5 μM), and VPA + Niclo treatment. (C) Determination of AcH3k9 protein level in H1299 and A549 cells by Western blotting. Indicates statistically significant differences in VPA, Niclosamide and VPA + Niclosamide combination treatment: *p < 0.05, **p < 0.01, ***p < 0.001. Data are presented as mean ± SD (*n* = 3). Densitometry was performed with ImageJ software and densitometric analysis of the observed band intensity measured relative to controls normalized to GAPDH and set to 1.0. GAPDH, glyceraldehyde 3‐phosphate dehydrogenase; SD, standard deviation.

Since VPA is an HDAC inhibitor, total histone H3 acetylation levels in lung cancer cells were analyzed by ELISA and Western blot method. When non‐toxic doses of VPA (100–250‐500 μM) were administered, total histone H3 acetylation increased, and the highest increase was observed at 500 μM. It was clearly observed that the combination treatment significantly increased total histone H3 acetylation in H1299 cells compared to VPA alone. The results of our previous study show that the same combination treatment also increased histone H3 acetylation in A549 cells (Figure 3A,B) [33]. Additionally, the change in Ac‐H3k9 protein levels caused by the combination of VPA and Niclo was evaluated in both A549 and H1299 cells. It was found that the VPA and Niclo combination treatment caused an increase in histone H3 Ac‐h3K9 protein levels in both A549 and H1299 cells compared to the control group, VPA and Niclo treatment (Figure 3C).

### 3.3 Effect of VPA + Niclosamide Combination on Cell Death

**FIGURE 4:**
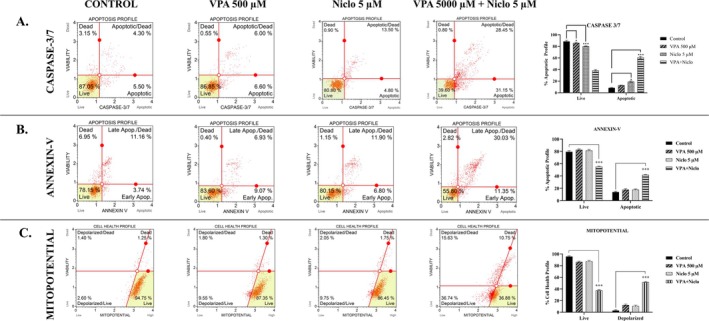
Evaluation of (A) Caspase 3/7 activity, (B) Annexin‐V ratios and (C) mitochondrial membrane potential ΔΨm change caused by Valproic acid (VPA), Niclosamide (Niclo) and VPA + Niclo combination treatment in H1299 cells. *Denotes statistically significant differences compared to untreated control: *(*p* < 0.05), **(*p* < 0.01), ***(*p* < 0.001). Data are presented as mean±SD (n=3).

In order to elucidate the cell death mode and the underlying mechanism, Annexin‐V ratio, caspase 3/7 activity, as well as mitochondrial membrane potential (MMP) of the cells receiving VPA + Niclo combination therapy compared to the control were evaluated. Flow cytometry analyzes showed that the combination of VPA + Niclo induced apoptosis in H1299 cells (Figure 4). Caspase 3 and caspase 7 are enzymes that play a key role in the process of apoptosis. They are often referred to as “executioner” caspases because they are responsible for the cleavage of many cellular proteins. They lead to the characteristic morphological and biochemical changes observed during apoptosis. According to the Flow cytometry analysis results, the percentage of apoptotic cells after VPA + Niclo combination treatment in H1299 cells increased from 4.30% to 28.465% compared to the control group (Figure 4A). In our previous study, after treatment with VPA, Niclo and VPA + Niclo combination in A549 cells, caspase 3/7 activity was 14.25% in the untreated group, while VPA, Niclo and combination treatment were found to be 13.8%, 13.5% and 50.02%, respectively [33]. Similarly, Annexin‐V activity (total apoptotic rate) increased to 30.03% in combination therapy, whereas VPA and Niclo treatments were 6.93% and 11.90%, respectively (Figure 4B). Following these results showing that VPA + Niclo treatment induced apoptosis in H1299 cells, MMP was evaluated to determine whether mitochondria contribute to apoptosis. As expected, the percentage of depolarized death cells increased significantly after combination therapy. While the percentage of depolarized dead cells was 1.80% and 2.05% in administration of VPA and Niclo alone, this rate increased above 15% after the combination treatment (Figure 4C). Similarly, MMP changed after combination treatment in A549 cells. The total percentages of mitochondrial depolarized cells in control were 6.6% and 7.05% for VPA and Niclo. In the combination treatment, the percentage of total mitochondrial depolarized cells in A549 cells was 62.15% [33].

### 3.4 Effect of VPA + Niclosamide Combination on Cell Cycle in Lung Cancer Cells

**FIGURE 5:**
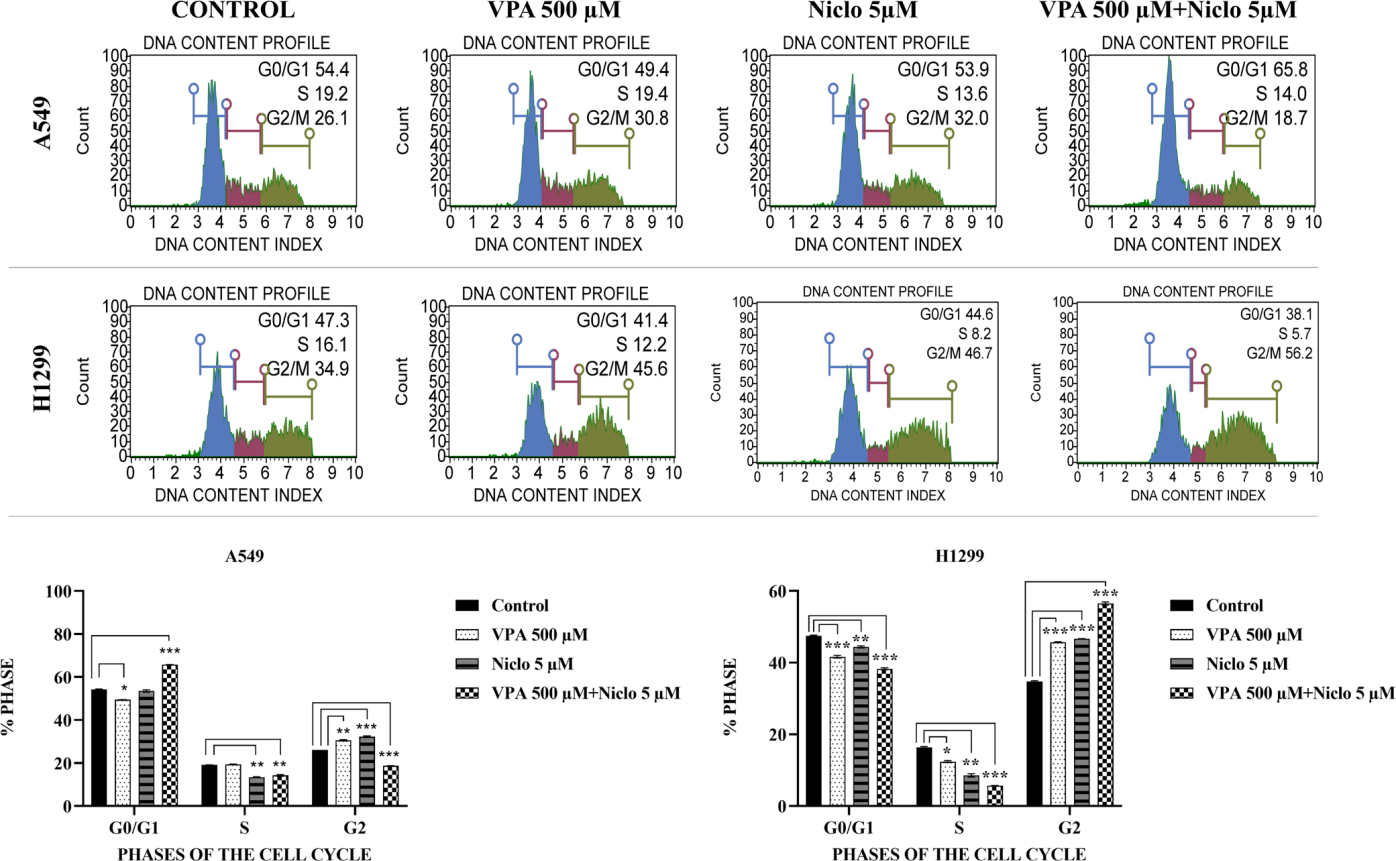
Histograms of percentage values obtained by evaluating the effect of Valproic acid (VPA), Niclosamide (Niclo) and VPA + Niclo combination treatments on the cell cycle in A549 and H1299 human lung cancer cells. *Denotes statistically significant differences compared to untreated control: *(*p* < 0.05), **(*p* < 0.01), ***(*p* < 0.001). Data are presented as mean±SD (n=3).

The distribution of cells in cell cycle phases was examined after VPA and Niclo applications alone and in combination in A549 and H1299 cells. In A549 cells, the cell cycle rates in the control group were G0/G1: 54.4%, S: 19.2%, G2/M: 26.1%. It was observed that after 500 μM VPA treatment alone, cell cycle rates changed to G0/G1: 49.4%, S: 19.4%, G2/M: 30.8%. It was observed that after only 5 μM Niclo treatment, the cell cycle ratios changed to G0/G1: 53.9%, S: 13.6%, G2/M: 32.0%. After VPA + Niclo combination treatment, cell cycle rates in A549 cells were observed to change to G0/G1: 65.8%, S: 14.0%, G2/M: 18.7% (Figure 5). In H1299 cells, the cell cycle rates in the control group were G0/G1: 47.3%, S: 16.1%, G2/M: 34.9%. It was observed that after 500 μM VPA treatment alone, cell cycle rates changed to G0/G1: 41.4%, S: 12.2%, G2/M: 45.6%. It was observed that after only 5 μM Niclo treatment, the cell cycle rates changed to G0/G1: 44.6%, S: 8.2%, G2/M: 46.7%. After VPA + Niclo combination treatment, cell cycle rates in H1299 cells were observed to change to G0/G1: 65.8%, S: 14.0%, G2/M: 18.7% (Figure 5).

### 3.5 Effect of VPA, Niclosamide, and Combination Treatment on Colony Formation in Lung Cancer Cells

**FIGURE 6:**
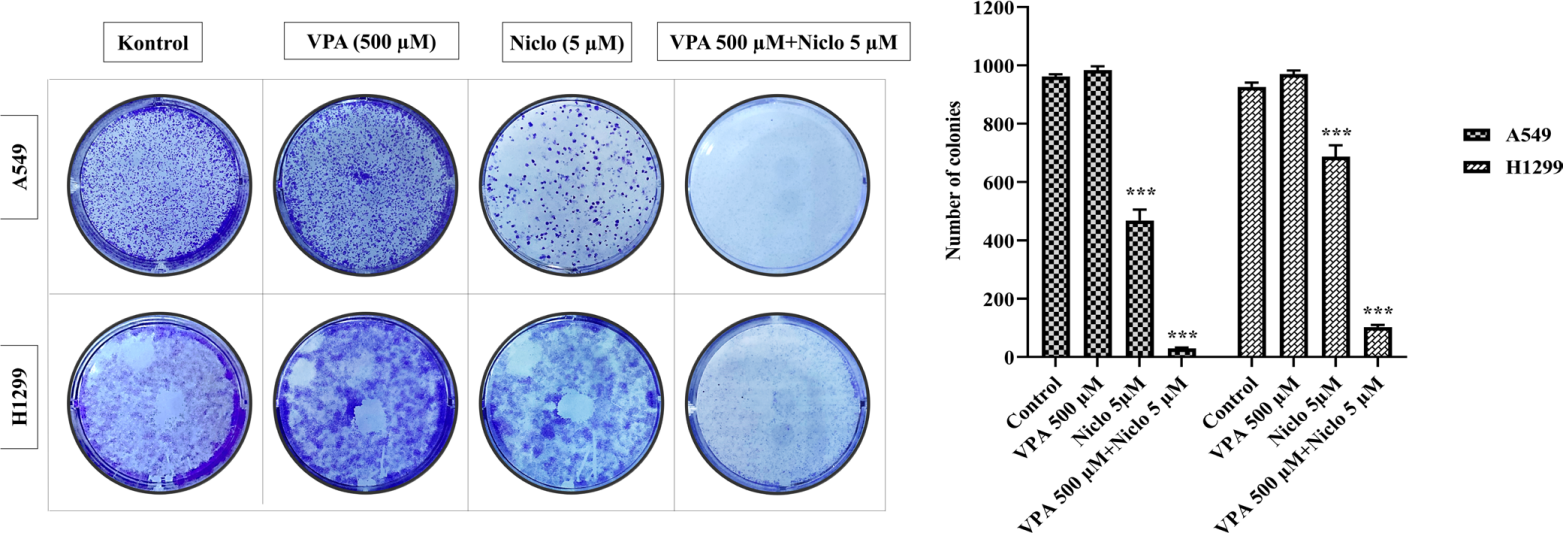
Effect of Valproic acid (VPA), Niclosamide (Niclo) and VPA + Niclo combination treatments on colony formation ability in A549 and H1299 human lung cancer cells. Examination of colony structures in A549 and H1299 cells stained with crystal violet. Shows statistically significant differences compared to control: ***p < 0.001. Data are presented as mean ± SD (*n* = 3). SD, standard deviation.

Following the application of VPA and Niclo alone and in combination, the colony formation method was applied to A549 and H1299 cells to evaluate the colony formation ability. A549 and H1299 cells were treated with VPA and Niclo alone and in combination for 48 h. At the end of the treatment period, the media in the environment was removed and fresh media was added. In the control group, growth medium was added until approximately 50 cells per colony were formed, and microscopic observation was made. The results obtained showed that the combination treatment caused a greater decrease in colony‐forming ability in both lung cancer cell lines compared to the control, compared to VPA and Niclo applications alone (Figure 6). As a result, it was observed that the ability of A549 and H1299 cells to form clones was lost in the presence of combination treatment, and the proliferative properties of the cells were inhibited after combination treatment.

### 3.6 Effect of VPA + Niclosamide Combination on Cell Migration Ability in Lung Cancer Cells

**FIGURE 7:**
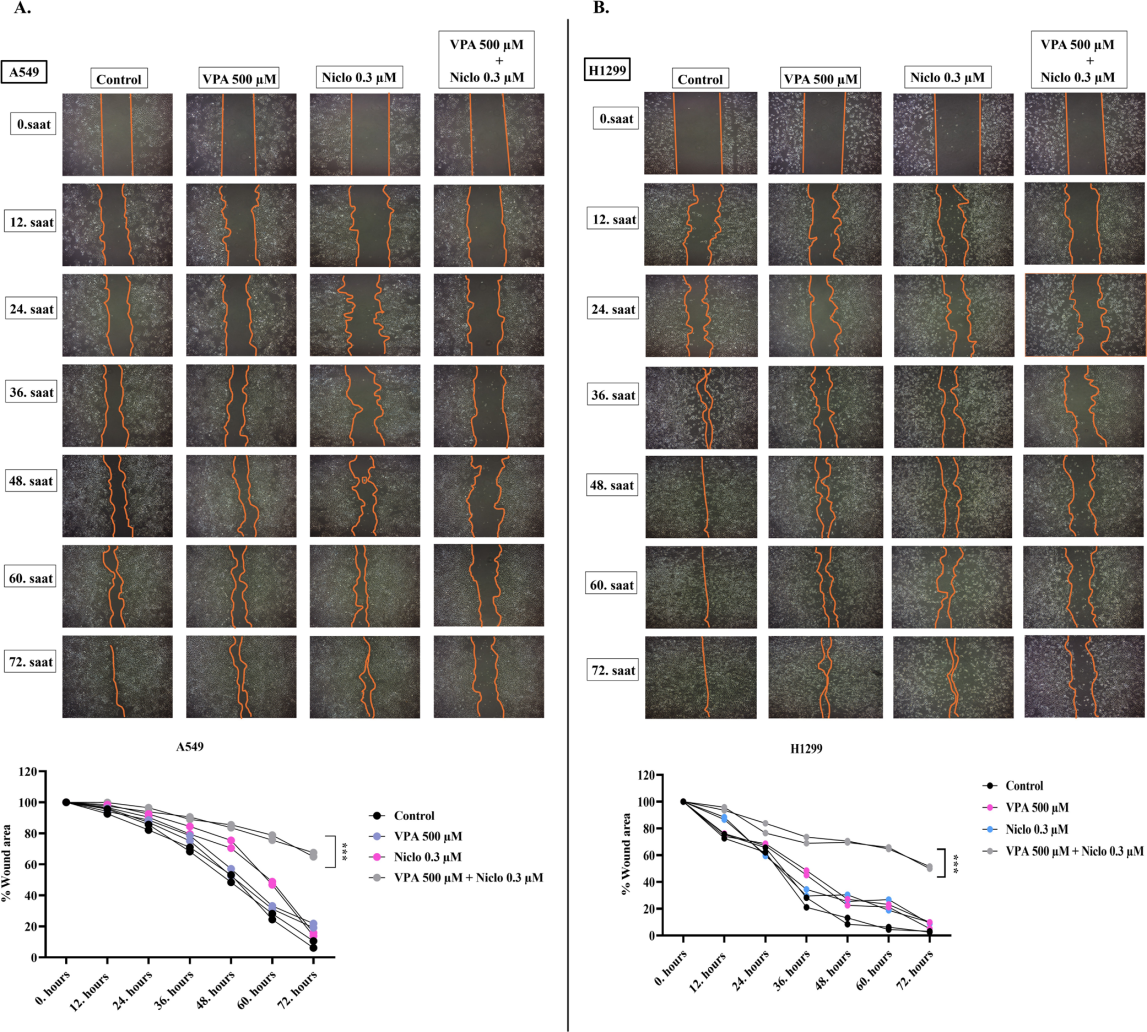
Wound healing assay in A549 (A) and H1299 (B) cells after treatment with VPA 500 μM, Niclo 0.3 μM and VPA + Niclo combination for 72 h. Combination treatment effect on A549 (A) and H1299 (B) cells migration evaluated by wound‐healing assay, the cell images were taken at 0, 12, 24, 36, 48, 60 and 72 h. The wound with of views was measured, and the healing width was calculated by wound with at 0 h time point minus wound with at 72 h time point and normalized by control. *Denotes significant difference between control group and combination‐treated cells (***p < 0.001).

Following the treatment of VPA and Niclo alone and in combination, the wound healing method was applied to evaluate the migration ability of A549 and H1299 cells. A549 and H1299 cells were treated with VPA and Niclo alone and in combination for 72 h. Wound healing rates were followed in the treatment and control groups for 72 h. The results obtained showed that the combination treatment limited the migration ability of the cells more compared to the control in both lung cancer cell lines compared to the VPA and Niclo alone (Figure 7). As a result, it was determined that the combination treatment showed a significant anti‐migration property on A549 and H1299 cells.

**FIGURE 8:**
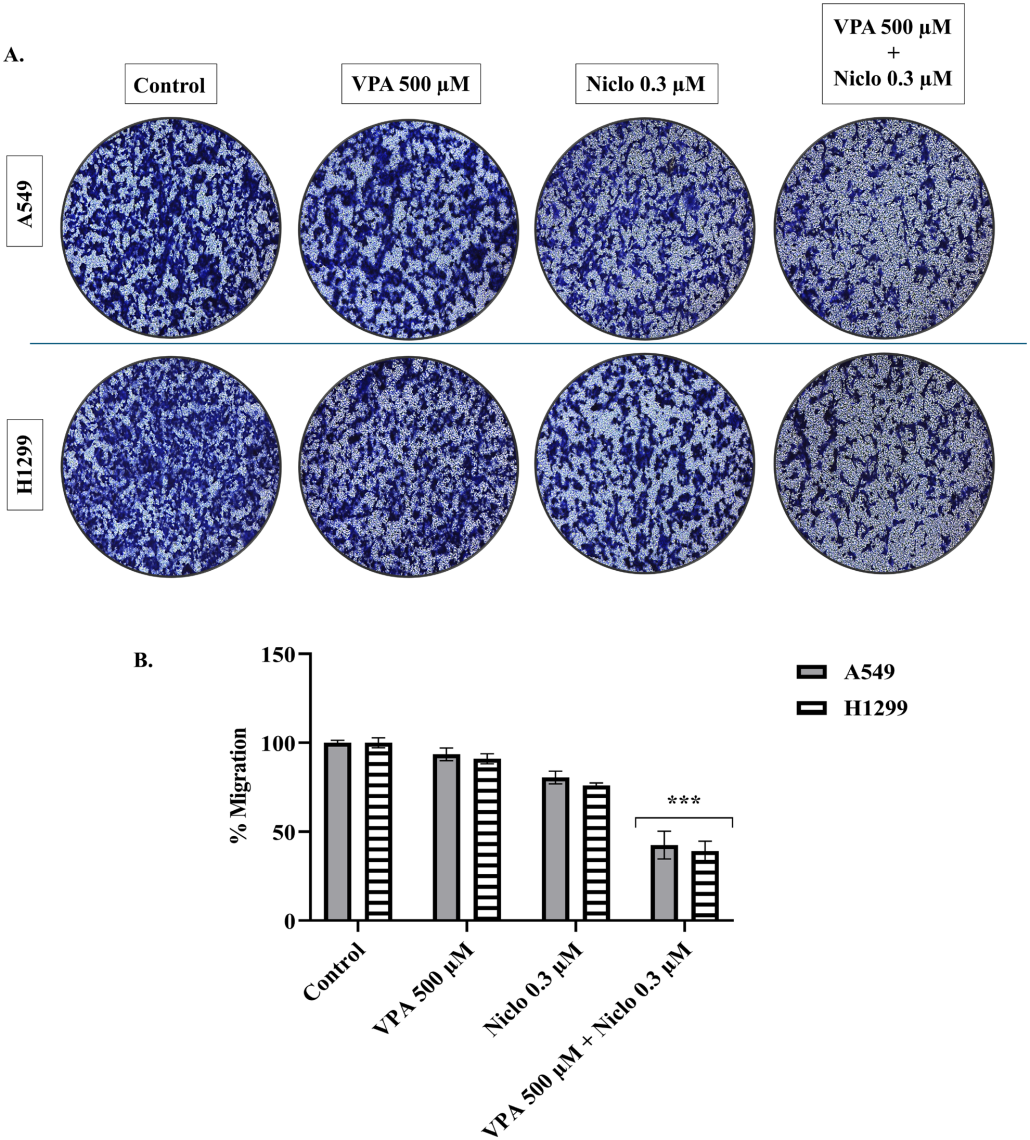
Transwell migration assay for VPA 500 μM, Niclo 0.3 μM and treatment of VPA + Niclo combination on cell migration in A549 and H1299 cells. A. Visualization of cell migration by phase contrast microscopy. B. Quantitative measurement of cell migration. Each data point represents the mean of 2 independent studies. *Denotes significant difference between control group and combination‐treated cells (***p < 0.001).

After the treatment of VPA and Niclo alone and in combination, a migration assay was performed using transwell chambers with 8 μm pore size to evaluate the migration ability of A549 and H1299 cells. When the obtained qualitative and quantitative results were evaluated, it was observed that the treatment of VPA and Niclo in combination, compared to the control, inhibited cell migration significantly (***p < 0.001) (Figure 8). These results indicate that the combined VPA and Niclo reduced the cell migration ability of A549 and H1299 cells.

### 3.7 Effect of VPA + Niclosamide Combination on EMT in Lung Cancer Cells

**FIGURE 9:**
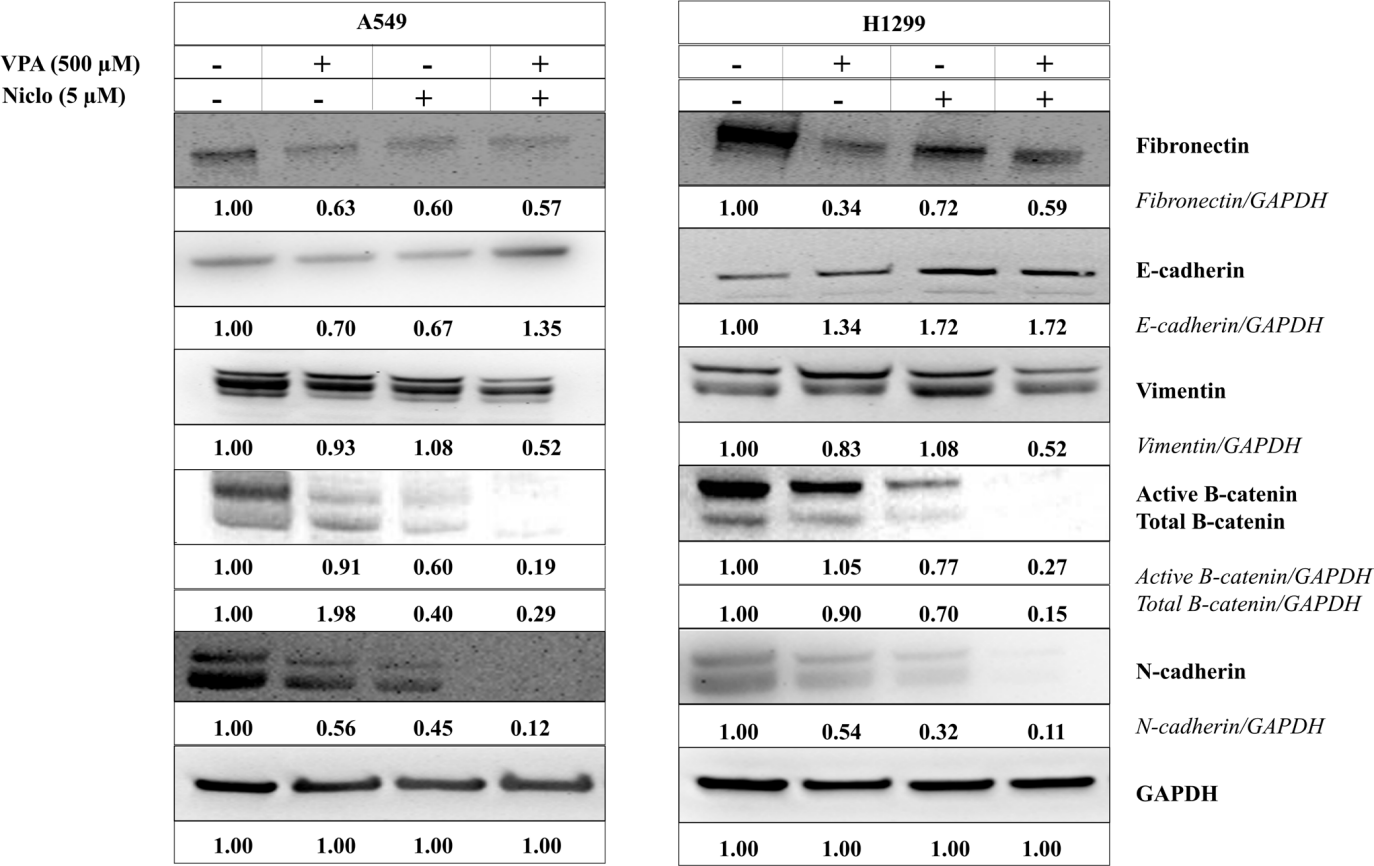
Western blot analysis of some EMT pathway proteins after application of Valproic acid (VPA) (500 μM), Niclosamide (Niclo, 5 μM), and their combination for 48 h in A549 **(A)** and H1299 **(B)** cells. Equal protein loading was confirmed by GAPDH. Densitometry was performed with ImageJ software and densitometric analysis of the intensity of observed bands normalized to GAPDH and quantified relative to controls set at 1.0. Data are presented as mean ± SD (*n* = 3). GAPDH, glyceraldehyde 3‐phosphate dehydrogenase; SD, standard deviation.

In order to determine the effect of VPA and Niclo combination on EMT in lung cancer, changes in the levels of markers specific to mesenchymal and epithelial cell characteristics were examined at both protein and gene levels. When the Western blot analysis results were examined, it was seen that combination therapy suppresses EMT in A549 and H1299 cells compared to VPA and Niclo. A significant increase in E‐cadherin, which constitutes epithelial cell characteristics, and a decrease in the protein levels of *β*‐catenin, N‐cadherin, Fibronectin, and Vimentin, which resulted in the disappearance of the mesenchymal character, have been detected (Figure 9).

**FIGURE 10:**
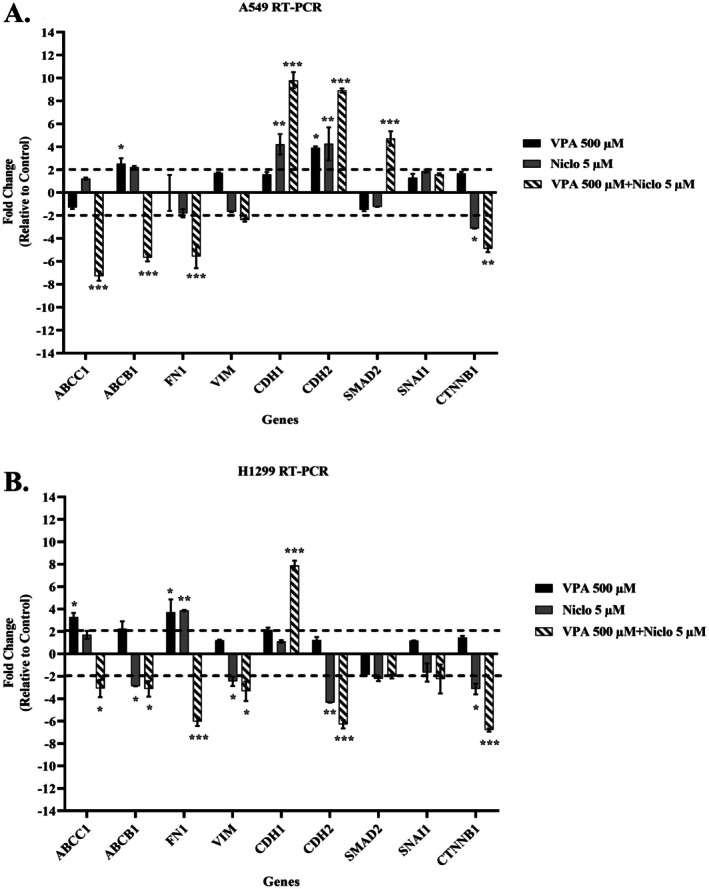
Changes in the expression profiles of some EMT genes determined by simultaneous PCR method after application of Valproic acid (VPA) (500 μM), Niclosamide (Niclo) (5 μM) and their combination for 48 h in (A) A549 and (B) H1299 cells. *Indicates statistically significant differences compared to control: *p < 0.05, **p < 0.01, ***p < 0.001. Data are presented as mean ± SD (*n* = 3). GAPDH, glyceraldehyde 3‐phosphate dehydrogenase; RT‐qPCR, quantitative real‐time polymerase chain reaction; SD, standard deviation.

When the results of RT‐qPCR analysis are examined, VPA + Niclo combination treatment reduced the expression levels of ABCB1 and ABCC1 genes, which are associated with EMT as well as multidrug resistance in both A549 and H1299 cells. Fibronectin (FN1) and Vimentin (VIM) gene expressions, which are associated with mesenchymal character, decreased after combination treatment. An increase was observed in the expression of the CDH1 gene (E‐Cadherin, Cadherin‐1), which is a marker of epithelial character. The expression levels of SNAIL and CTTNB1 genes, which are activated during the EMT process, decreased after combination treatment, especially in H1299 cells (Figure 10A,B).

## 4 Discussion

Lung cancer is one of the leading causes of cancer‐related deaths in the world and poses a challenging global health burden. The clinical success of treatment in lung cancer is not very promising due to the diagnosis at an advanced stage, limited treatment tools, and the development of recurrence and drug resistance [38]. In addition, a comprehensive understanding of the underlying molecular mechanisms that cause lung cancer progression is required. Comprehensive molecular characterization of lung cancer has expanded our understanding of the origins of cells and molecular pathways affected in each of the lung cancer subtypes [39]. Chemotherapy or combined chemotherapy protocols are created according to the lung cancer cell type, taking into account the effect/side effect profiles. In preclinical research, the combined use of various drugs that affect different molecular pathways of the cancer cell is common in chemotherapeutic treatments of lung cancer. Recent cancer research has shown that VPA, known as a histone deacetylase inhibitor (HDAC inhibitors, HDACi, HDIs) can combine with agents targeting different molecular pathways, leading to different mechanisms of action [40, 41, 42, 43]. Accordingly, in this study, the effects of the combination of VPA, a histone deacetylase inhibitor, and Niclo, which is used as an anthelmintic drug in the clinic, on apoptosis, cell cycle control, and EMT in lung cancer cells were evaluated.

In the current study, the combination of VPA + Niclo exhibited stronger growth inhibitory effects in lung cancer than applying these compounds alone. There are many studies in which VPA and Niclo compounds are combined with different compounds showing anticancer activity in lung cancer. Arsenic trioxide, which has anti‐cancer effects in various solid tumors as well as hematological malignancies, has been shown to strongly inhibit cell growth when combined with VPA and applied to lung cancer cells [27]. It is known that the combination of cisplatin, a well‐known cytostatic agent, and VPA causes anti‐proliferative effects in lung cancer through the MTT test, similar to the results of many combination studies in the literature [44, 45, 46]. It was thought that the combination application of the EGF receptor inhibitor Erlotinib and Niclo to Erlotinib‐resistant lung cancer cells could restore Erlotinib sensitivity in these cells [47]. In an old study conducted by our group, it was shown that the combination of anthracycline‐based chemotherapy (FEC: 5‐fluorouracil+epirubicin+cyclophosphamide) and VPA increased the cytotoxic effect of FEC in breast cancer cells [48]. As a result, it is thought that combination treatments can gain an advantage against tumor cells by targeting multiple cellular pathways.

VPA is known to inhibit histone deacetylases (HDACs) at therapeutic concentrations [43, 49]. One study found that VPA and its analogs inhibited multiple HDACs with a characteristic order of action in vitro. These analogs also induce hyperacetylation of core histones H3 and H4 in intact cells, with a degree of effect parallel to the inhibition in vitro [50]. In a study conducted with cancer stem cell‐like cells, it was observed that VPA at relatively lower doses (2.5 and 5 mM) prevented mammosphere formation and increased acetylation of histone H3 [21]. Additionally, improved results in tumor inhibition were obtained when VPA was combined with chemotherapeutic agents in vitro and in vivo [41]. In this study, it was determined that the VPA + Niclo combination treatment caused a greater increase in H3 acetylation levels in lung cancer cells than VPA and Niclo treatments alone. As a result, the effect of HDACi can be increased by combining VPA with various chemotherapy agents that have a direct anticancer effect.

There are many studies showing that Niclo, an FDA‐approved anthelmintic drug, causes apoptosis in cancer cells [51, 52, 53]. In one study, to confirm the hypothesis that Niclo‐induced apoptosis in thyroid cancer cells (TPC‐1) may occur via the mitochondrial apoptotic pathway, mitochondrial membrane potential (ΔΨm) was measured using Rh123 staining. It was concluded that Niclo caused ΔΨm loss of TPC‐1 cells in a concentration‐dependent manner, and especially the ΔΨm loss of the 20 μM‐treated group was 54.1% higher than the control group [54]. In a different study, it was reported that Niclo significantly inhibited proliferation in CT26 cells, a colorectal cancer cell line, causing loss of mitochondrial membrane potential [55]. In this study, as a result of the combination of VPA with Niclo, it is observed that apoptosis is stimulated by the disruption of mitochondrial membrane potential. It was determined that combination treatment caused a greater increase in apoptotic cell rates than VPA and Niclo applications alone.

There is increasing evidence that epigenetic changes such as histone acetylation and promoter methylation play important roles in regulating cell cycle‐related gene expression [56]. HDACi are a promising class of drugs that function as antiproliferative agents by promoting differentiation, as well as inducing cell cycle arrest [57]. VPA can inhibit the proliferation of hepatocellular carcinoma cells by inducing G1 phase arrest and cell apoptosis [58]. Similarly, Niclo was found to be cytotoxic, arresting the cell cycle, and increasing the effects of chemotherapeutic drugs in combination studies [58, 59, 60]. In another study, Niclo was found to induce S and G2/M phase arrest in osteosarcoma cell lines (U2OS and MG‐63) [53]. It was also revealed that Niclo induction of p21 and G1 arrest of the cell cycle suppressed the STAT3 signaling pathway and inhibited cell proliferation in esophageal adenocarcinoma cells (BE3) and esophageal squamous cell carcinoma cells (CE48T and CE81T). Additionally, combination therapy of Niclo and chemotherapeutic drugs selectively reduced the dose requirement of chemotherapeutic drugs to achieve IC_50_ efficacy [61]. Studies with different cell lines revealed that these two chemicals induce apoptosis in cells by stopping the cell cycle. In this study, it was observed that the combination of VPA and Niclo decreased cell retention in S phase in lung cancer cell lines from 19.2% to 14.0% in A549 cells compared to the control, and from 16.1% to 5.7% in H1299 cells. Apart from this, in the combination treatment in A549 cells, there was an increase from 54.4% to 65.8% in the G0/G1 phase, and a decrease from 47.3% to 38.1% in H1299 cells. In the G2/M phase, a decrease from 26.1% to 18.7% was observed in A549 cells and an increase from 34.9% to 56.2% in H1299 cells. Accordingly, after combination therapy, G1 phase retention increased in A549, while S phase retention decreased. In H1299 cells, retention in G1 phase and S phase decreased, while retention in G2/M phase increased. The retention of cells in G0/G1 phase was interpreted as these agents causing a decrease in cell growth rate. The arrest of the cell cycle in the G2/M phase suggests that repairing intracellular DNA damage is difficult and cells enter apoptosis.

One of the most frequently used methods in cancer research is the method based on colony formation ability. Clonogenic testing or colony formation testing is a cell survival test that tracks the ability of a single cell to grow into a colony [36]. In order to evaluate the cytotoxic or genotoxic effects of potential agents during cell growth, cancer cells capable of forming colonies after treatment can be compared with the control group, and the amount of dose‐dependent change can be evaluated qualitatively or quantitatively. Tian and colleagues examined post‐treatment colony‐forming abilities in MCF‐7 and EUFA423 breast cancer cell lines to confirm the cell sensitivity caused by the combination of VPA and hydroxyurea (HU). The results of the clonogenic survival assay showed that the survival fraction in cells treated with the combination of VPA and HU was significantly reduced compared to other groups [62]. In a different study supporting this research topic, it was observed that VPA sensitized three glioma cells, namely U251, LN229, and SNB19, to luteolin, a natural anticancer agent of flavonoid origin, by suppressing cell viability, colony formation, and migration [63]. Based on this information, as a result of the colony formation ability test we performed in this study, it was determined that VPA + Niclo combination treatment significantly prevented colony formation compared to the control. Moreover, the proliferative activities of both lung cancer cells were suppressed compared to VPA and Niclo treatment alone.

EMT, as first described in embryonic development, results in the transition of epithelial cells to cells with a mesenchymal phenotype defined by prototypic markers such as E‐cadherin and vimentin. Activation of genes and proteins that are markers of EMT increases cancer cell motility for mass migration in both cell clusters and individual cells, thereby promoting invasion and spread [64]. E‐cadherin is a type of cell adhesion molecule that is primarily found in epithelial cells. It plays a crucial role in maintaining the integrity and cohesion of epithelial tissues by mediating cell–cell adhesion. During the process of EMT, epithelial cells lose their characteristic morphology and acquire a mesenchymal phenotype. This process is accompanied by a decrease in E‐cadherin expression and concomitant increases in the expression of mesenchymal markers such as *β*‐catenin, fibronectin, vimentin, and N‐cadherin [65]. The loss of E‐cadherin expression is considered a hallmark of EMT, as it results in the disruption of cell–cell adhesion and the acquisition of migratory and invasive properties by epithelial cells. E‐cadherin downregulation is thought to be a critical step in the initiation of EMT, as it allows for the dissociation of epithelial cells and their subsequent migration to new sites in the body. Overall, the role of E‐cadherin in EMT is to maintain the integrity and cohesion of epithelial tissues and prevent the acquisition of mesenchymal characteristics by epithelial cells [66]. Therefore, the increase in E‐cadherin levels after VPA + Niclo treatment indicates that combination therapy may be useful for inhibiting the EMT process in lung cancer cells. In contrast to E‐cadherin, *β*‐catenin, fibronectin, vimentin, and N‐cadherin are four key proteins that are upregulated during the process of EMT, which is a critical step in tumor progression and metastasis. Vimentin is a type III intermediate filament protein and N‐cadherin is a type of cell adhesion molecule that is normally expressed in mesenchymal cells. During EMT, the expression of *β*‐catenin, fibronectin, vimentin, and N‐cadherin is upregulated in epithelial cells, which gives them a more mesenchymal‐like characteristics and allows them to adopt a migratory and invasive phenotype. Vimentin expression is also associated with resistance to apoptosis and increased tumor cell survival, and N‐cadherin can promote cell survival, resistance to apoptosis, and activate several signaling pathways that are involved in tumor progression [67]. Therefore, in the current study, decreased expression levels of *β*‐catenin, fibronectin, vimentin, and N‐cadherin after VPA + Niclo combination treatment may play a critical role in inhibiting mesenchymal phenotype acquisition and reducing cell invasion ability, inhibiting tumor cell survival and resistance to apoptosis. In addition, wound healing and transwell migration assays also showed that combined VPA and Niclo treatment significantly inhibited cell migration. Studies have indicated that HDAC inhibitors such as VPA have the potential to regulate the EMT process in different types of cancer. It has been observed that VPA can stimulate the expression of E‐cadherin in cancer cells and inhibit the expression of mesenchymal markers, including N‐cadherin and vimentin. For example, Yang and Wang found that VPA inhibited the invasion and metastasis of glioma cells through upregulation of E‐cadherin and downregulation of vimentin and N‐cadherin [68]. Similar to the findings of our study, another study reported that VPA treatment decreased N‐cadherin and vimentin levels and increased E‐cadherin levels in kidney cancer cells. It has also been described that inhibition of EMT by VPA is associated with a decrease in SMAD4 [24]. Combination drug studies with VPA show that it limits cell movement and reduces metastatic capacity [40, 46]. It has been found that Niclo inhibits the most important markers involved in the EMT signaling cascade stimulated by TGF‐*β*, such as N‐cadherin, Snail, and Slug [69]. Apart from these, it was presented in another study that Niclo inhibited the most important markers such as N‐cadherin, Snail, and Slug, which are involved in the EMT signaling cascade stimulated by TGF‐*β* [69]. In summary, compared with previous studies, our findings show that VPA and Niclo are effective on EMT markers, but VPA + Niclo combination treatment has a much greater potential in inhibiting the EMT process in lung cancer cells.

## 5 Conclusion

In conclusion, our study shows that the combination of VPA + Niclo is a promising approach that may have a synergistic effect to inhibit EMT in lung cancer cells. Overall, available evidence suggests that HDAC inhibitors such as VPA have the potential to regulate EMT in lung cancer cells, especially when used in combination with other drugs. However, further preclinical and clinical studies are needed to fully understand their potential benefits and limitations in EMT regulation and lung cancer treatment.

## Conflicts of Interest

The authors declare no conflicts of interest.

## Supporting information


**Figure S1.** Percent viability graphs of A549 and H1299 cells treated with VPA (500 μM) + Niclo (0.07–5 μM) for 48 h. ATP assay was performed as cell viability assays. *Denotes statistically significant differences compared to untreated control: *p < 0.05, **p < 0.01, ***p < 0.001. Data are presented as mean ± SD (*n* = 3).

## Data Availability

The data that support the findings of this study are available from the corresponding author upon reasonable request.
