# Supplementary figures and images for: A New Treatment Strategy for Lung Cancer With HDAC and Wnt/ β ‐Catenin Pathway Inhibitors
Source: IUBMB Life. 2025 Jul 12;77(7):e70037. doi: 10.1002/iub.70037 (PMC12254719; doi:10.1002/iub.70037)

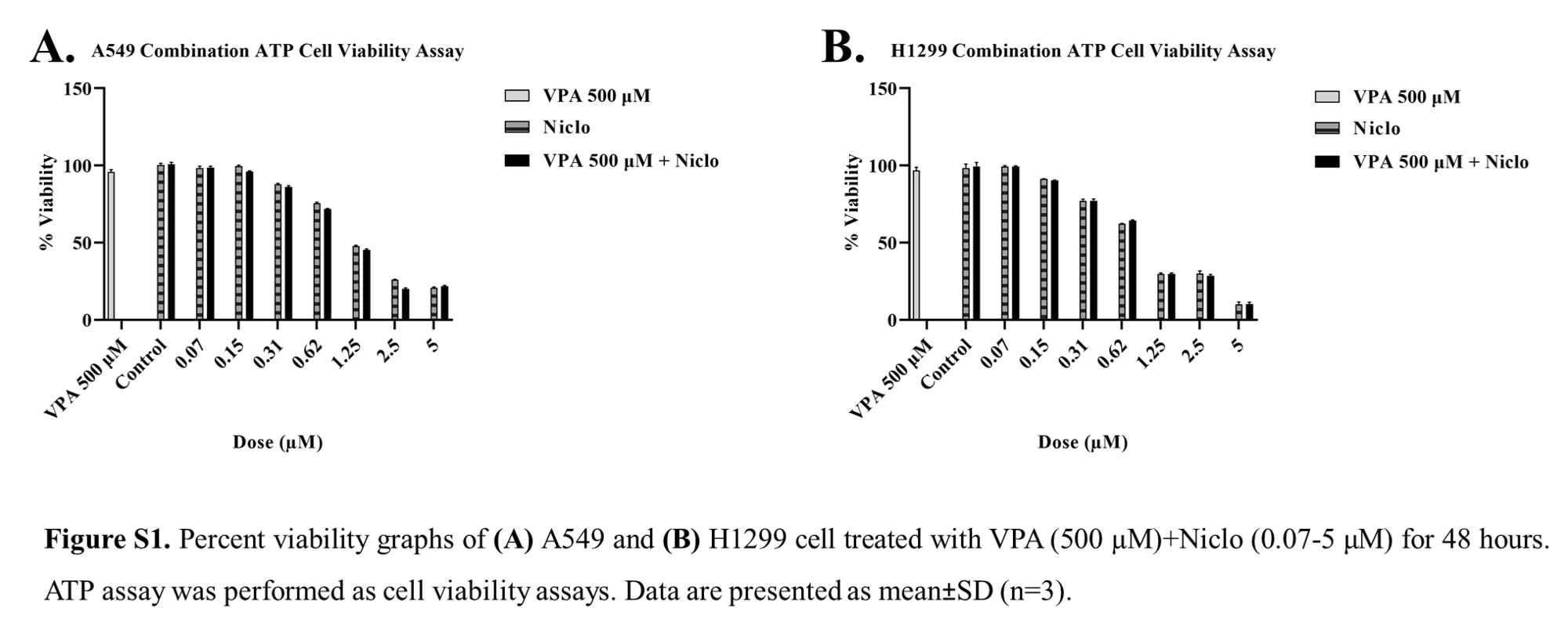

Supplement: Supplementary file 1 — Figure S1. Percent viability graphs of A549 and H1299 cells treated with VPA (500 μM) + Niclo (0.07–5 μM) for 48 h. ATP assay was performed as cell viability assays. *Denotes statistically significant differences compared to untreated control: *p < 0.05, **p < 0.01, ***p < 0.001. Data are presented as mean ± SD (n = 3). [file IUB-77-0-s001.tif]
